# Chiral Pesticides in Soil and Water and Exchange with the Atmosphere

**DOI:** 10.1100/tsw.2002.109

**Published:** 2002-02-08

**Authors:** Terry F. Bidleman, Andi D. Leone, Renee L. Falconer, Tom Harner, Liisa M.M. Jantunen, Karin Wiberg, Paul A. Helm, Miriam L. Diamond, Binh Loo

**Affiliations:** Meteorological Service of Canada, 4905 Dufferin Street, Downsview, ON M3H 5T4, Canada; 885 North Hubbard Road, Lowellville, OH 44436, USA; Department of Chemistry, Chatham College, Woodland Road, Pittsburgh, PA 15232, USA; Department of Chemistry, Environmental Chemistry, Umeå University, SE-901 87 Umeå, Sweden; Department of Chemical Engineering and Applied Chemistry, University of Toronto, Toronto, ON M5S 3E5, Canada; Department of Geography, University of Toronto, Toronto, ON, M5S 3G3, Canada; Hawaii Department of Agriculture, 1428 South King Street, Honolulu, HI 96814, USA

**Keywords:** air-soil, air-water, chiral, enantiomer, organochlorine pesticide

## Abstract

The enantiomers of chiral pesticides are often metabolised at different rates in soil and water, leading to nonracemic residues. This paper reviews enantioselective metabolism of organochlorine pesticides (OCPs) in soil and water, and the use of enantiomers to follow transport and fate processes. Residues of chiral OCPs and their metabolites are frequently nonracemic in soil, although exceptions occur in which the OCPs are racemic. In soils where enantioselective degradation and/or metabolite formation has taken place, some OCPs usually show the same degradation preference — e.g., depletion of (+)trans-chlordane (TC) and (–)*cis*-chlordane (CC), and enrichment of the metabolite (+)heptachlor exo-epoxide (HEPX). The selectivity is ambivalent for other chemicals; preferential loss of either (+) or (–)o,p’-DDT and enrichment of either (+) or (–)oxychlordane (OXY) occurs in different soils. Nonracemic OCPs are found in air samples collected above soil which contains nonracemic residues. The enantiomer profiles of chlordanes in ambient air suggests that most chlordane in northern Alabama air comes from racemic sources (e.g., termiticide emissions), whereas a mixture of racemic and nonracemic (volatilisation from soil) sources supplies chlordane to air in the Great Lakes region. Chlordanes and HEPX are also nonracemic in arctic air, probably the result of soil emissions from lower latitudes. The (+) enantiomer of *α*-hexachlorocyclohexane (*α*-HCH) is preferentially metabolised in the Arctic Ocean, arctic lakes and watersheds, the North American Great Lakes, and the Baltic Sea. In some marine regions (the Bering and Chukchi Seas, parts of the North Sea) the preference is reversed and (–)*α*-HCH is depleted. Volatilisation from seas and large lakes can be traced by the appearance of nonracemic *α*-HCH in the air boundary layer above the water. Estimates of microbial degradation rates for *α*-HCH in the eastern Arctic Ocean and an arctic lake have been made from the enantiomer fractions (EFs) and mass balance in the water column. Apparent pseudo first-order rate constants in the eastern Arctic Ocean are 0.12 year^-1^ for (+)*α*-HCH, 0.030 year-1 for (–)*α*-HCH, and 0.037 year^-1^ for achiral *Υ*-HCH. These rate constants are 3–10 times greater than those for basic hydrolysis in seawater. Microbial breakdown may compete with advective outflow for long-term removal of HCHs from the Arctic Ocean. Rate constants estimated for the arctic lake are about 3–8 times greater than those in the ocean.

## INTRODUCTION

Many organochlorine pesticides (OCPs) are global contaminants, dispersed primarily through the atmosphere. Decades after being deregistered in Canada and the U.S., OCPs are still routinely found in air and precipitation from the Great Lakes region[1,2,3,4,5] and the Arctic[6,7,8]. International treaties and regional action plans have been established to ban or phase out persistent organic pollutants (POPs)[9]. Twelve POPs, eight of which are OCPs (aldrin, dieldrin, DDT, hexachlorobenzene, chlordane, heptachlor, mirex, and toxaphene), were recently banned under the United Nations Environmental Program (UNEP) Global POPs Protocol[10].

A new approach to investigating pesticide transport and fate in the environment uses chiral analytical methods to determine individual enantiomers. Several classes of pesticides have members that are chiral: organochlorines, organophosphates, pyrethroids, and herbicides. Chiral OCPs include o,p′-DDT, α-hexachlorocyclohexane (α-HCH), heptachlor (HEPT), several components of technical chlordane and technical toxaphene, and the metabolites oxychlordane (OXY), heptachlor exo-epoxide (HEPX), and o,p′-DDD.

Although a few modern chiral pesticides are now manufactured as single-enantiomer products, most are racemates. Enantiomers have the same physical and chemical properties; therefore, abiotic transport processes (leaching, volatilisation, atmospheric deposition) and reactions (hydrolysis, photolysis) are not likely to be enantioselective. (Some exceptions are discussed in the Conclusions section.) Metabolism by microorganisms in water and soil often proceeds enantioselectively, leading to nonracemic residues.

In this article, we review applications of pesticide enantiomers for tracing transport and transformation processes. We specifically focus on air-soil and air-water exchanges and selective degradation pathways in the physical environment. Details of our own studies and analytical methods are given in primary publications[11,12,13,14,15,16,17,18,19,20,21]. Chiral methods have also been applied in several recent food web and bioaccumulation studies[22,23,24,25,26,27,28,29,30 and references cited therein], although these are not discussed here. Reviews of chiral analytical methods and enantioselective metabolism[31,32] of xenobiotics and a book devoted to these subjects[33] have been published.

## CHIRAL ORGANOCHLORINE PESTICIDE RESIDUES IN SOILS

Quantitative measurements of OCP residues in soils are useful for several purposes: assessing the reservoir of pesticides held in the terrestrial environment, determining bioaccumulation in plants and terrestrial biota (e.g., earthworms), and modeling soil-air exchange. Chiral OCPs in soils have been measured at several locations in North America. Residues have been determined in agricultural soils of Alabama[12], the Midwestern U.S. (Ohio, Indiana, Illinois, and western Pennsylvania)[13,14] and the Fraser Valley of British Columbia, Canada[15,16]. Compounds sought were o,p´-DDT (and other achiral DDTs), chlordanes, HEPT, OXY, HEPX, α-HCH, and, in Alabama, toxaphene (though no chiral studies were done for toxaphene). In Connecticut, chlordanes only were determined in agricultural, home lawn and garden, and house foundation soils[34,35,36]. Results from ten soil samples collected from farms on Molokai, Hawaii, and analysed using previously published methods[13,15] are first reported here.

Residue data from these studies are summarized in Table 1 as ranges, arithmetic means (AMs), or geometric means (GMs), depending on the information given in the original publication. Although there have been other quantitative determinations of OCPs in soils, only the locations where enantiomeric measurements have also been made are included in Table 1. Concentrations in the soils varied by several orders of magnitude, even within a relatively small region. Log normal distributions and GMs are probably the best measures of variability and central value[13,15]; however, some reports give neither GMs nor the individual sample data to calculate them. OCP residues did not correlate with either the percentage organic carbon for most soils in Alabama (0.3–3.5% organic carbon)[12] or the percentage in the Midwestern U.S. (0.7–7.6% organic carbon)[13]; however, one Midwestern soil with 33% organic carbon contained exceptionally high levels of OCPs. In British Columbia, OCP concentrations increased from loamy sand (1.0–1.8% organic matter), to silt loams (3.7–6.5% organic matter), to muck soils (27–56% organic matter)[37].

Ratios of p,p´-DDT to the metabolite p,p´-DDE (DDT/DDE) are often used to roughly estimate the age of the residues, since the proportion of p,p´-DDE tends to increase with time. This ratio in the individual soil samples varied from 0.5–1.4 in Hawaii to 0.5–1.5 in Alabama[12] and to 0.5–6.6 in the Midwestern U.S.[13]. DDT/DDE ratios derived from geometric mean concentrations were 0.88 in Hawaii, 0.84 in Alabama, and 1.25 in the Midwestern U.S. Chiral OCPs were reported in soil samples from the U.K. that had been archived between 1968 and 1990[38]. The samples came from experimental plots; one was amended with sewage sludge in 1968 and the other was an unamended control site. Residues of hexachlorobenzene, DDE, and dieldrin were slightly higher and significantly different ($p > 0.05$) in the sludged plot compared to the control plot. Ratios of DDT/DDE varied from 0.14–1.0, but surprisingly showed no trend with time.

Chlordane residues in soils representing three land use classes in Connecticut were determined: agricultural, home lawn and garden, and house foundation[34,36]. Total chlordane (*trans*-chlordane + *cis*-chlordane + *trans*-nonachlor = TC + CC + TN) was highest in foundation soils (mean = 7,180 ng/g dry wt.), followed by residential (330 ng/g) and agricultural (142 ng/g) soils. A detailed study was also carried out on one lawn site located at the Connecticut Agricultural Experiment Station where chlordane had been applied once in 1960 and had remained under continuous grass cover until 1998, when the soil samples were collected prior to tilling the site for planting crops. Total chlordane averaged 7,689 ng/g dry wt. Within the treatment site, the chlordane components were in the proportion CC ≥ TC > TN, and this order was also found for house foundation soils where residues exceeded 5,000 ng/g dry wt. Chlordane residues in soil samples taken ~2–40 m outside the treated site were approximately 1% of those inside, and proportions of the three chlordane components showed considerable variation. The investigators also showed that weathered chlordane residues from the experimental site were accumulated in several crops via root uptake and deposition of atmospheric residues onto leaves[35].

*Trans*-chlordane is thought to be metabolised more rapidly than *cis*-chlordane in soils[36]; thus, the ratio of TC/CC should be an indicator of such metabolism. Differences in the proportions of chlordane isomers are found among soils, as noted above for sites in Connecticut. The ratios of TC/CC in the U.K. soils ranged from 0.6–2.9[38]. TC/CC ratios in the individual Hawaiian soils ranged from 4.1–6.5, and averaged 5.3 when calculated from GM concentrations. Similar statistics were 0.54–1.7 (mean = 0.94) in Alabama[12], and 0.33–6.0 (mean = 1.1) in the Midwestern U.S.[13]. The TC/CC ratio in technical chlordane is 0.95–1.18[34,39]. Overall, Hawaiian soils contained a greater proportion of TC than soils from the other regions. This might be due to the use of heptachlor in Hawaiian agriculture, since technical heptachlor is contaminated with chlordane isomers[40].

**TABLE 1**
**Chiral Organochlorine Pesticides in Soils, ng/g Dry Weight**

| Compound | Location | Year | Samples[a] | Range or AM[b] | S.D. | GM[b] | Ref. |
|---|---|---|---|---|---|---|---|
| DDT[c] | Alabama | 1996 | 38 | 54 | 38 | 17 | 12 |
| | Midwestern U.S. | 1995–1996 | 34 | <0.5–11,800 | | 9.6 | 13 |
| | Hawaii | 1999 | 10 | 1,540 | 1,200 | 1,080 | This work |
| | British Columbia | 1989 | | | | | 15,37 |
| | Muck soils | | 4 | 2,984–7,162 | | | |
| | Silt loam soils | | 4 | 194–1,275 | | | |
| | U.K.[d] | 1968–1990 | 6 | 1.4–3.2 | | | 38 |
| | U.K.[e] | 1968–1990 | 6 | 2.0–9.2 | | | 38 |
| Chlordane[f] | Alabama | 1996 | 32 | 1.9 | 1.1 | 0.6 | 12 |
| | Midwestern U.S. | 1995–1996 | 32 | <0.2–847 | | 1.3 | 13 |
| | Connecticut | 1997–1999 | | | | | 34–35 |
| | Experimental site | | 35 | 7,689 | 4,340 | | |
| | Agricultural soils | | 53 | 142 | 120 | | |
| | Lawn/garden soils | | 34 | 330 | 987 | | |
| | House foundation soils | | 45 | 7,180 | 14,296 | | |
| | Hawaii | 1999 | 10 | 110 | 71 | 88 | This work |
| | British Columbia | 1989 | | | | | 15,37 |
| | Muck soils | | 4 | 284–1,943 | | | |
| | Silt loam soils | | 4 | 0.6–338 | | | |
| | U.K.[d] | 1968–1990 | 6 | 0.17–0.54 | | | 38 |
| | U.K.[e] | 1968–1990 | 6 | 0.09–0.81 | | | 38 |
| Heptachlor | Alabama | 1996 | 3 | 0.04 | 0.02 | 0.04 | 12 |
| | Midwest U.S. | 1995–1996 | 16 | <0.04–56 | | 0.11 | 13 |
| | Hawaii | 1999 | 10 | 9.4 | 8.7 | 2.7 | This work |
| | British Columbia | 1989 | | | | | 15,37 |
| | Muck soils | | 4 | 37–278 | | | |
| | Silt loam soils | | 4 | 0.16–1.0 | | | |
| Heptachlor exo-epoxide | Alabama | 1996 | 12 | 0.19 | 0.14 | 0.10 | 12 |
| | Midwest U.S.[g] | 1995–1996 | 27 | <0.04–121 | | 0.49 | 13 |
| | Hawaii[g] | 1989 | 4 | 16–174 | | 19 | This work |
| | British Columbia | 1989 | | | | | 15.37 |
| | Muck soils | | 4 | 85–390 | | | |
| | Silt loam soils | | 4 | 0.05–36 | | | |
| α-HCH | Alabama | 1996 | 24 | 0.10 | 0.12 | 0.05 | 12 |
| | Midwest U.S. | 1995–1996 | 12 | <0.04–1.2 | | 0.09 | 13 |
| | British Columbia | 1989 | | | | | 15.37 |
| | Muck soils | | 4 | 10–259 | | | |
| | Silt loam soils | | 4 | 0.26–2.3 | | | |
| | U.K.[d] | 1968–1990 | 6 | 0.04–0.37 | | | 38 |
| | U.K.[e] | 1968–1990 | 6 | 0.06–0.24 | | | 38 |
| Toxaphene | Alabama | 1996 | 39 | 285 | 390 | 84 | 12 |

[a] Total sites: Alabama = 39, Midwestern U.S. = 40, Connecticut = 86, Hawaii = 10, British Columbia = 12, U.K. = 1.
[b] AM, GM = arithmetic, geometric means.
[c] Some or all of: p,p'-DDT, o,p'-DDT, p,p'-DDE, o,p'-DDE, p,p'-DDD, o,p'-DDD.
[d] Archived soils from one control (unsludged) site; only the range is given because the samples are from different years.
[e] Archived soils from one site, treated with sewage sludge in 1968.
[f] Some or all of *cis*-chlordane, *trans*-chlordane, and *trans*-nonachlor.
[g] Includes oxychlordane.

## ENANTIOSELECTIVE METABOLISM OF ORGANOCHLORINE PESTICIDES IN SOILS

Enantioselective metabolism of chiral pesticides often takes place in soils, leading to the presence of nonracemic residues. Resolution of the individual enantiomers of OCPs can be accomplished by capillary gas chromatography (GC) on derivatised cyclodextrin columns with detection by mass spectrometry[13,14,15]. Results are usually presented as the enantiomer ratio (ER), defined as the concentration of (+)/(–) enantiomers, or the first/second eluting enantiomer when the optical signs are not known[31,32,33]. Here we use the enantiomer fraction, EF = (+)/[(+) + (–)], which is preferred over ER[41,42].

EF values for chiral OCPs and metabolites in soils of different regions are given in Table 2. In our work, replicate injection of racemic standards (EF = 0.50) yielded standard deviations of 0.015 or less[13,15]. For parent OCPs, EF values less than 0.50 and greater than 0.50 indicate preferential metabolism of the (+) and (–) enantiomer, respectively. In the case of metabolites OXY and HEPX, EF $\neq$ 0.50 could arise from preferential formation of the (+) or (–) product from the parent compound and/or selective degradation of the opposite enantiomer of the metabolite. The EF values in Table 2 suggest regional differences in the metabolism of OCPs, although only a few soils from each region have been analysed by chiral methods.

Enantioselective metabolism in most agricultural soils favoured degradation of (+)TC and (–)CC. Exceptions were British Columbia, where racemic chlordanes were found in the six soils examined[15], and at one farm in Connecticut[36]. Two other Connecticut farms, along with a test plot at the Connecticut Agricultural Experiment Station, contained nonracemic chlordanes. Soils near house foundations in Connecticut contained very high chlordane residues which were racemic. The mean EF of TC in Midwestern U.S. agricultural soils (0.41)[13] was significantly different ($p < 0.01$) from the mean EFs in Alabama (0.48)[21] and Hawaii (0.47)[this work]. Differences in EFs of CC among the three regions (0.52–0.55) were not significant ($p > 0.05$). Eitzer et al.[36] found a strong (negative) correlation between the ERs of TC and CC in Connecticut soils, and demonstrated that a similar correlation existed in the data of Aigner et al.[13] for Midwestern U.S. soils.

Technical chlordane was applied to the test plot in Connecticut once in 1960 and the plot lay under grass cover until it was tilled in 1998. Investigation of the site before and after tilling showed no significant difference in total chlordane residues, but significant differences ($p < 0.05$) were found in the ERs of TC and CC, and in the ratios TC/TN and CC/TN. Soil tillage appeared to enhance enantioselective degradation. The U.K. experimental plots showed significantly ($p < 0.05$) nonracemic EFs for both TC (EF = 0.40–0.46) and CC (EF = 0.52–0.57), although there was neither significant difference between sludged and unsludged soils nor a trend with time from 1968 to 1990[38].

In some studies, EFs of the chlordane component MC5 were also determined. The elution order of (+) and (–) enantiomers are not known for MC5, so the EF was calculated as A1/(A1 + A2), where A1 and A2 refer to the areas of peaks eluting first and second on a Betadex-120 or Gammadex-120 chiral phase column[13,14,15,21,36]. In soils showing enantioselective degradation, the first-eluting enantiomer of MC5 was preferentially depleted (EF < 0.50).

Soils show a strong preference for (+)HEPX accumulation. The EFs in Alabama (0.67)[21] and the Midwestern U.S. (0.71)[13] were different from the EF in Hawaii (0.55) ($p < 0.01$), and the EFs in the Midwestern U.S. and British Columbia (0.56)[15] were different from each other ($p < 0.01$). EFs in Alabama and British Columbia were not significantly different ($p > 0.05$) due to the small number of samples in each set.

Residues of α-HCH were high enough for chiral analysis in British Columbia, the U.K., a few soils in Alabama, and one Midwestern soil. The α-HCH in three British Columbia silt loams was racemic (EF range = 0.49–0.50), whereas selective breakdown of (–)α-HCH was found in three muck soils (EF range = 0.55–0.58). The same selectivity was found for one soil sample near a former HCH factory in Germany (EF = 0.52)[43]. The EF averaged 0.50 in seven Alabama soils and was 0.49 in the one Midwestern soil. EFs in the U.K. soils ranged from 0.48–0.52 and were generally not significantly different *($p$ > 0.05)* from racemic in either sludged or unsludged treatments[38].

Residues of OXY and o,p′-DDT showed selective accumulation of the (+) enantiomer in some soils and the (−) enantiomer in others (Table 2). In the case of OXY, the EFs in soil may be the net result of selective metabolism of (+)TC and (−)CC to OXY enantiomers with these respective signs. Eitzer et al.[36] found a correlation ($r^2 = 0.68$) between the CC/TC ratio and percentage of OXY in Connecticut soils, demonstrating more rapid conversion of TC rather than CC to OXY. Reasons for the ambivalence of o,p′-DDT are not known.

Soils from Alabama[12] and other southern U.S. states[44] contain residues of toxaphene resulting from former usage on cotton, soybeans, and other crops. So far, no chiral studies have been done on these residues, but the chromatographic pattern in soil[44] and ambient air[39] indicates selective depletion of certain labile congeners, notably B8-531 (2,2,3-exo,5-endo,6-exo,8,9,10-octachlorobornane) and B8-806/809 (2,2,5-endo,6-exo,8,8,9,10 and 2,2,5-endo,6-exo,8,9,9,10-octachlorobornanes). Vetter and Maruya[45] found enantioselective depletion of certain toxaphene congeners in the lower food web from a toxaphene-contaminated salt marsh in Georgia, although the ones mentioned above in soils were not investigated.

Enantioselective degradation of the herbicide dichlorprop after field application of the racemic mixture was followed by capillary electrophoresis using the chiral reagent heptakis(2,3,6-tri-O-methyl)-β-cyclodextrin[46]. Breakdown of the herbicide occurred almost completely within a month. The S-(−) enantiomer degraded significantly faster than the R-(+) enantiomer, in contrast to the results of a laboratory study with marine microorganisms which degraded the R-(+) form exclusively.

Lewis et al.[47] studied the effects of simulated global warming, deforestation, and nutrient addition on the enantioselective degradation in soil of the organophosphorus insecticide ruelene and the herbicides dichlorprop and methyl dichlorprop. Methyl dichlorprop was demethylated quickly ($t_{1/2}$ ~0.7 days), whereas degradation of parent dichlorprop and ruelene proceeded only after lag times of ~6 months. Simulated global warming was investigated by raising the temperature of North American and Norwegian soils by 5$^o$C over a 4- to 7-year period. Soil samples were then collected and incubated with pesticides in the laboratory. Neither heating nor addition of inorganic nutrients affected the enantioselectivity for methyl dichlorprop demethylation. Addition of organic nutrients (beef broth) caused soils which preferentially demethylated the (+) enantiomer to strongly prefer the (−) enantiomer in soils from North America and Brazil, but not in those from Norway, where soil degradation already favoured the (−) enantiomer. In Brazil, deforestation caused soils to shift from preferentially removing the (−) enantiomer of ruelene to exclusively degrading the (+) enantiomer. Soil warming in Norway caused soils to shift from all samples degrading (+) ruelene to 22% preferring (−) ruelene.

Lewis et al.[47] suggested that microbial enantioselectivity towards environmental pollutants is controlled by the activation of metabolically quiescent microbial populations or the induction of enantiomer-specific enzymes. Changes in enantioselectivity could be due to different groups of related microbial genotypes being activitated under different environmental conditions. This is also suggested by the increased enantioselective degradation of TC and CC after tilling the soil at the Connecticut test plot[36]. Reductive dechlorination of o,p′-DDT was investigated in the laboratory using the aquatic plant elodea *(Elodea canadensis)* and the terrestrial plant kudzu *(Pueraria thunbergiana),* a partially purified fraction of an *Elodia* extract, the porphyrin hematin, and human hemoglobin[48]. Although all of these systems degraded o,p′-DDT with formation of o,p′-DDD, none was enantioselective.

**TABLE 2**
**Enantiomer Fractions (EF) of OCPs in Soils[a,b]**

| Compound | Location | Samples | Range | AM[c] | S.D. | Ref. |
|---|---|---|---|---|---|---|
| o,p'-DDT | Alabama | 30 | 0.41-0.57 | 0.50 | 0.036 | 21 |
| | Midwest U.S. | 16 | 0.43-0.54 | 0.49 | 0.038 | 13 |
| | Hawaii | 9 | 0.45-0.50 | 0.48 | 0.014 | this work |
| | British Columbia | 6 | 0.45-0.51 | 0.50 | 0.022 | 15 |
| | U.K.[d] | 10 | 0.48-0.57 | 0.51 | 0.027 | 38 |
| Trans-chlordane | Alabama | 11 | 0.46-0.50 | 0.48 | 0.012 | 21 |
| | Midwest U.S. | 23 | 0.32-0.48 | 0.41 | 0.040 | 13 |
| | Connecticut | | | | | 36 |
| | experimental site | 35 | | 0.46 | 0.02[e] | |
| | agricultural soils | 13 | 0.41-0.50 | 0.47 | 0.02 | |
| | house foundation soils | 15 | | 0.49 | 0.01 | |
| | Hawaii | 10 | 0.44-0.49 | 0.46 | 0.018 | this work |
| | British Columbia | 6 | 0.49-0.50 | 0.50 | 0.003 | 15 |
| | U.K.[d] | 11 | 0.40-0.46 | 0.43 | 0.019 | 38 |
| Cis-chlordane | Alabama | 11 | 0.48-0.56 | 0.53 | 0.022 | 21 |
| | Midwest U.S. | 23 | 0.50-0.61 | 0.55 | 0.029 | 13 |
| | Connecticut | | | | | 36 |
| | experimental site | 35 | | 0.54 | 0.03[e] | |
| | agricultural soils | 13 | 0.51-0.55 | 0.53 | 0.01 | |
| | house foundation soils | 15 | | 0.50 | 0.005 | |
| | Hawaii | 10 | 0.49-0.53 | 0.52 | 0.011 | this work |
| | British Columbia | 6 | 0.49-0.50 | 0.50 | 0.005 | 15 |
| | U.K.[d] | | 0.52-0.57 | 0.54 | 0.017 | 38 |
| Oxychlordane | Alabama | 3 | 0.55-0.60 | 0.54 | 0.023 | 21 |
| | Midwest U.S. | 17 | 0.40-0.62 | 0.52 | 0.066 | 13 |
| | Hawaii | 9 | 0.52-0.65 | 0.60 | 0.049 | this work |
| | British Columbia | 4 | 0.37-0.46 | 0.41 | 0.039 | 15 |
| Heptachlor exo-epoxide | Alabama | 4 | 0.50-0.76 | 0.67 | 0.119 | 21 |
| | Midwest U.S. | 14 | 0.54-0.88 | 0.71 | 0.087 | 13 |
| | Hawaii | 10 | 0.53-0.57 | 0.55 | 0.013 | this work |
| | British Columbia | 4 | 0.55-0.58 | 0.56 | 0.016 | 15 |
| α-HCH | Alabama | 7 | 0.48-0.53 | 0.50 | 0.013 | 21 |
| | Midwest U.S. | 1 | | 0.49 | | 13 |
| | British Columbia | 6 | 0.49-0.58 | 0.53 | 0.037 | 15 |
| | U.K.[d] | 11 | 0.48-0.52 | 0.50 | 0.010 | 38 |
| | Germany | 1 | | 0.52 | | 43 |

[a] EF = (+)/[(+) + (–)] enantiomers.
[b] See text for compound abbreviations.
[c] AM = arithmetic mean.
[d] One location, sludge-treated and control plots, followed from 1968–1990.
[e] ERs instead of EFs were reported for Connecticut samples. Standard deviations for EFs calculated here were assumed to be half those reported for the ERs.

## ENANTIOSELECTIVE METABOLISM OF PESTICIDES IN WATER

Most investigations of chiral pesticides in the aquatic environment have been for α-HCH. EFs in these studies are summarised in Table 3. Early work showed enantioselective degradation of α-HCH in the North Sea[49,50,51]. The degradation preference was ambivalent, with some regions of the North Sea showing depletion of the (+) enantiomer, and others showing the (–)[49].

Enantioselective degradation of α-HCH is accompanied by formation of two chiral β-pentachlorocyclohexenes (β-PCCHs), whereas degradation of achiral γ-HCH produces two chiral

**TABLE 3**
**Enantioselective Degradation of α-HCH in Oceans and Lakes**

| Location | Year | Range of EFs | Ref. |
|---|---|---|---|
| *Marine* | | | |
| Bering Sea | 1993 | 0.52–0.53 | 17 |
| Chukchi Sea | 1993–1994 | 0.51–0.55 | 17,18 |
| Western Arctic Ocean | | | |
| Surface–50 m | 1992–1994 | 0.41–0.48 | 17,18,52 |
| Surface | 1998 | 0.45 | 26 |
| 300–2200 m | 1994 | 0.11–0.44 | 17 |
| Eastern Arctic Ocean | 1996 | | 19 |
| Surface–50 m | | 0.42–0.48 | |
| 200–1000 m | | 0.11–0.37 | |
| North Sea | 1987 | 0.44–0.54 | 49–51 |
| | 1994–1995 | 0.45–0.48 | 76 |
| Baltic Sea | 1987 | 0.44–0.48 | 50 |
| | 1997–1998 | 0.44–0.46 | 20 |
| South Atlantic Ocean | 1997 | 0.49–0.52 | 54 |
| Southern Ocean | 1997 | 0.48–0.51 | 54 |
| *Freshwater — Canadian Arctic and Subarctic* | | | |
| Lakes, Eastern Arctic | 1992–1998 | 0.36–0.43 | 52,56,57, 59 |
| Lakes, Yukon | 1993 | 0.24–0.47 | 58,59 |
| Streams and wetlands, Eastern Arctic | 1992–1998 | 0.26–0.50 | 52,56,57, 59 |
| *Freshwater — Canadian Temperate* | | | |
| Small Ontario lakes | 1999 | 0.47–0.51 | 59 |
| Ontario streams and wetlands | 1999 | 0.39–0.47 | 59 |
| Great Lakes | 1993–1998 | 0.46–0.47 | 11,59 |

γ-PCCHs. The relative proportions of these isomers and their respective enantiomers in the aquatic environment are controlled by rates of formation by dehydrochlorination of the HCHs and degradation of the PCCHs[50]. Investigations in Skagerrak[50] and the southern Baltic Sea[20,50] showed uniform depletion of (+)α-HCH. Selective degradation of (−)α-HCH was found in the Bering and Chukchi Seas[17], but (+)α-HCH was degraded preferentially in the western[17,18,26,52] and eastern[19,53] Arctic Ocean. The α-HCH residues were slightly nonracemic in surface water of the South Atlantic and Southern oceans between South Africa and Antarctica, with differences in enantiomer preference among regions[54].

When racemic α-HCH was added to a culture of microorganisms isolated from aerobic marine sediment, (+)α-HCH was preferentially dehydrochlorinated to form nonracemic β-PCCH[50]. Anaerobic breakdown of α-HCH in active sewage sludge also favoured the (+) enantiomer. A slower rate of degradation was found in sterilized sludge with no chiral preference[55].

The EF of α-HCH decreases with depth in the western[17] and eastern[19,53] Arctic Ocean, due to preferential loss of (+)α-HCH in the deeper, older water layers (Fig. 1). Degradation rates of γ-HCH and the two enantiomers of α-HCH in the eastern Arctic Ocean were estimated *in situ* from measured vertical profiles of concentration and enantiomer composition (for α-HCH) in the water column between 200 and 800 m, and the estimated total loss of HCHs from that water mass

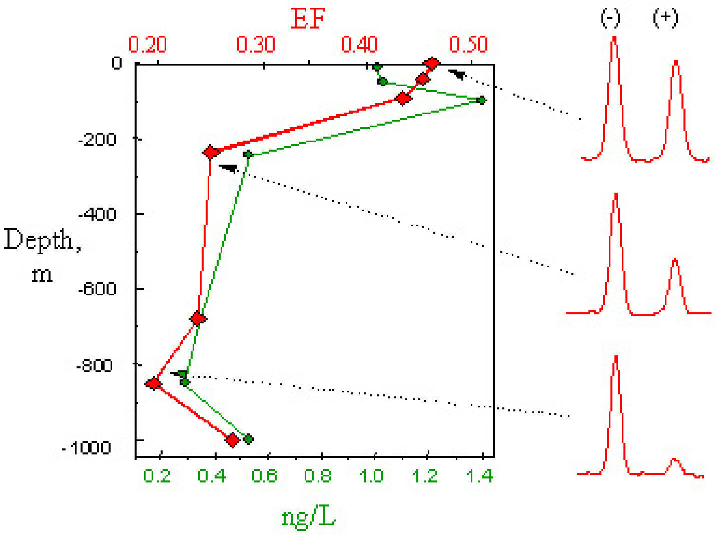

**FIGURE 1.** Enantiomer chromatograms (right) and vertical profiles of concentration (thin line, circles) and EF (thick line, diamonds) with depth (left) of α-HCH in the eastern Arctic Ocean. Note the preferential loss of (+)α-HCH in the deeper, older water layers. Data from Harner et al.[19,53].

from 1979 to 1996[19,53]. Pseudo first-order microbial degradation rates were 0.12 $year^{-1}$ for (+)α-HCH, 0.030 $year^{-1}$ for (–)α-HCH, and 0.037 $year^{-1}$ for γ-HCH[19]. These were 3–10 times faster than rates of basic hydrolysis in seawater. As a result, microbial degradation may account for a third of the total annual HCH loss from the Arctic Ocean, competing with advective outflow[53].

The (+) enantiomer of α-HCH was selectively depleted in Canadian arctic lakes, streams, and wetlands[52,56,57,58,59] and in the North American Great Lakes[11,59]. Factors influencing the enantioselective degradation in lakes and wetlands have been investigated[57,59]. Preferential degradation of (+)α-HCH was extensive in arctic watersheds and wetlands resulting in EF = 0.27–0.47[56,57,58,59]. Arctic lakes also exhibited low EF (0.24–0.47)[52,56,57,58,59]. By contrast, α-HCH in small lakes from temperate regions of Canada was more nearly racemic (EF = 0.47–0.51), and the range of temperate wetland and stream EFs (0.39–0.47) indicated less enantioselectivity than in arctic wetlands[59]. Law et al.[59] put forward the hypothesis that enantioselective degradation is optimised by maximal contact between the α-HCH and sediment substrates in nutrient-poor water where oligotrophic bacteria might act as biofilms.

Comparisons were made of α-HCH degradation in one arctic lake (Amituk Lake) and its inflowing streams[57]. Approximately 7% of the α-HCH was enantioselectively degraded in the watershed prior to entering the lake. A mass balance in the lake showed that over-winter loss amounted to 34–63% of the water column inventory. Sedimentation was negligible, outflow in winter was zero, and hydrolysis was estimated to account for only 2% of the loss. The remaining loss was attributed to microbial degradation, with an apparent pseudo first-order rate constant of 0.48–1.13 $year^{-1}$. This value is about 3–8 times greater than the combined microbial degradation rate for the two α-HCH enantiomers in the Arctic Ocean[19]. Most of the over-winter degradation within the lake appeared to be due to nonenantioselective processes.

The enantiomer composition of chlordane and HEPX in Arctic Ocean surface water was determined in a 1994 study (10–14 samples)[18]. TC and CC were both racemic (EF = 0.50 ± 0.006 and 0.50 ± 0.008, respectively). As in soils, HEPX was enriched in the (+) enantiomer (EF = 0.62 ± 0.011).

Studies have been carried out on the enantioselective degradation and interconversion of enantiomers of the herbicides mecoprop and dichlorprop in water[60,61]. Only the R-(+)enantiomers are herbicidally active, and these were degraded more slowly than the inactive S-

(−)enantiomers[60,61]. In Switzerland, only R-(+)mecoprop is registered as a herbicide, but interconversion of enantiomers in the environment led to the S-(−)enantiomer being detected in Swiss lakes[62]. R-(+) and S-(−)mecoprop in some lakes[62] and a polluted aquifer[63] may have also resulted from inputs of racemic mecoprop from nonagricultural sources.

Metolachlor is a herbicide which contains four stereoisomers due to two chiral elements — an asymmetric carbon and a chiral axis — in its structure. Metolachlor was first used as a racemate, but since 1997 has been partially replaced by S-enriched metolachlor which contains about 90% 1´S-isomers[64]. Surface water from Swiss lakes sampled prior to 1998 contained racemic metolachlor, but a clear excess of the 1´S-isomers was found in 1998 and 1999, indicating contributions of the newly released nonracemic herbicide[64]. Concentrations and enantiomer compositions in surface and deep waters were followed over 2 years and a computer simulation was used to estimate that 55% (1998) and 90% (1999) of the racemic metolachlor had been substituted by S-metolachlor. The authors pointed out that there was no marked difference in metolachlor concentrations over this period; thus, the switch of metolachlor products could not have been deduced by concentration measurements.

Bromcyclen is a bicycloheptene containing six chlorines and one bromine, used against ectoparasites in Europe. Water samples taken in the River Stör, a tributary of the Elbe in Germany, contained racemic bromcyclen. The bromcyclen in fish from the Stör was nonracemic and differed between trout, which showed depletion of the (−) enantiomer, and bream, in which depletion of the (+) enantiomer was found[65].

## EXCHANGE WITH SOIL AND WATER

### Air-Soil Exchange

The enantiomer signatures of nonracemic pesticides in soil and water are preserved upon volatilisation, providing useful tracers for following emissions. Pesticides in air are subject to only abiotic removal processes which are nonenantioselective, such as photolysis, hydroxyl radical attack, and atmospheric deposition. Enantiomers have the same physicochemical properties, and thus EFs are not subject to fractionation effects as isomer- and parent/metabolite ratios are. For these reasons, EF values in air are conservative and can carry information about the sources of pesticides[66,67].

Volatilisation of α-HCH and HEPX into the air above an agricultural field was investigated at a farm in the Fraser Valley, British Columbia[16], where (−)α-HCH was depleted and (+)HEPX enriched in the soil. Fig. 2 shows vertical profiles of OCP concentration and EFs in the air above the soil. Although concentrations decline with height for both compounds, there is a distinct difference in the behaviour of the EFs. Those for α-HCH also decrease with height, while the HEPX EFs are relatively invariant. For α-HCH, the nonracemic emissions from the soil may be diluted with more nearly racemic α-HCH from distant transport. Background α-HCH in the atmosphere has an EF close to 0.50[11,14]. In the case of HEPX, the soil of this particular field may be the main source to the overlying air, or the background air has a similar EF value. Ambient air samples from the southern U.S. and the Great Lakes region contained nonracemic HEPX, with EF values ranging from 0.60 to 0.67[66,68,69].

Chlordane was used in the U.S. until 1978 for agriculture, ornamentals, home lawns and gardens, and termite control. After 1983, its only permitted use was as a termiticide, and all uses were cancelled in 1988[70]. The chlordane in ambient air today may come from volatilisation of residues in soils, emission of termiticides from treated buildings, and long-range transport from countries where chlordane is still used.

Ambient air samples from the Great Lakes region contained nonracemic TC and CC[14,66,67,69], whereas nearly racemic chlordanes were found in the air of Alabama[39] and

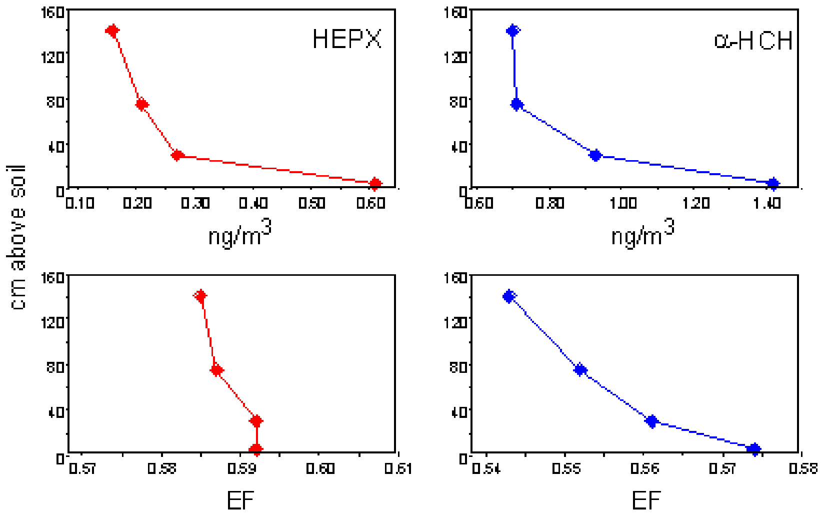

**FIGURE 2.** Profiles of OCP concentration (upper) and EF (lower) in air above an agricultural field in the Fraser Valley, British Columbia. Data from Finizio et al.[16].

the city of Columbia, SC[39,66,67]. Residues of TC and CC in agricultural soils in Alabama[21], the Midwestern U.S.[13], and one farm in the U.K.[38] were nonracemic (Table 2). Indoor air concentrations of chlordanes in Alabama and South Carolina[39] and the Midwestern U.S.[71] greatly exceeded those in ambient air, and the TC and CC were racemic. Fig. 3 shows the chromatographic profile of nonracemic chlordanes in an Ohio soil. EF values for this soil, Columbia urban air, and Great Lakes air are also shown[67].

Studies at six farms in the Midwestern U.S. were carried out by collecting paired soil and overlying air samples[14]. Both soil and air contained nonracemic chlordanes, HEPX, OXY, and o,p′-DDT. Vertical profiles of chlordane concentrations and EFs were obtained at one farm. Although concentrations decreased from 10 to 175 cm above the soil, indicating soil-to-air exchange, the EFs were invariant as seen for HEPX in British Columbia (Fig. 2)[16]. EFs of chlordanes in air-above-soil samples at the six farms averaged 0.423 ± 0.024 for TC and 0.526 ± 0.017 for CC. Means for 13 ambient air samples, collected at a height of 80 cm in rural but nonagricultural situations, were 0.481 ± 0.005 for TC and 0.509 ± 0.005 for CC. These are intermediate of air-above-soil EFs and the racemic EFs found for indoor air[71].

Results of these investigations suggest that both racemic and nonracemic sources contribute to observed chlordane EFs in Great Lakes air[66,67,69]. In contrast, racemic sources appear to dominate chlordane inputs to urban air in South Carolina and ambient air in Alabama[39,67].

Background air in the northern hemisphere is likely to be influenced by long-range transport of pesticide residues emitted from contaminated soils[72]. Air samples collected from the North American and Eurasian Arctic in 1994–1998 and from the west coast of Sweden in 1998 contained nonracemic HEPX with mean EFs of 0.663–0.696[73]. This is most likely the result of enantioselective metabolism of heptachlor in soils and subsequent emission of HEPX[68]. TC (EF = 0.451–0.478) and sometimes CC (EF = 0.503–0.513) were also nonracemic in these air samples (Fig. 4). On the other hand, TC and CC in surface water sampled under the ice cap in the Canadian Arctic in 1994 were racemic[18], possibly due to residues that date from earlier decades when chlordane was in common use.

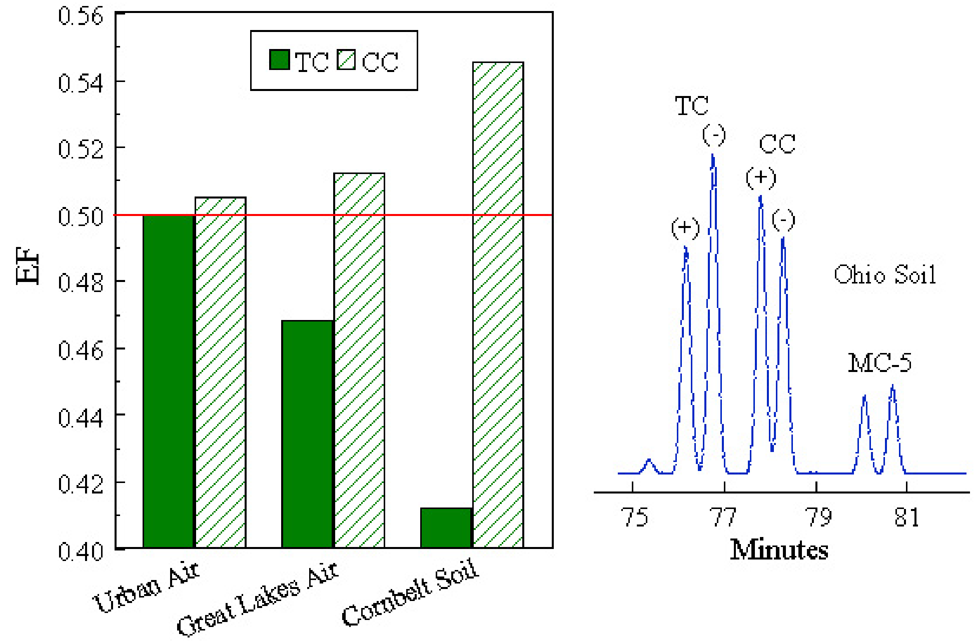

**FIGURE 3.** Right: chromatographic separation of enantiomers of TC, CC, and another chlordane (MC5) in an Ohio soil sample. Left: EF values for urban air (Columbia, SC), Great Lakes air, and average soil from the Midwestern U.S. cornbelt. Reproduced (modified) from Bidleman et al.[67].

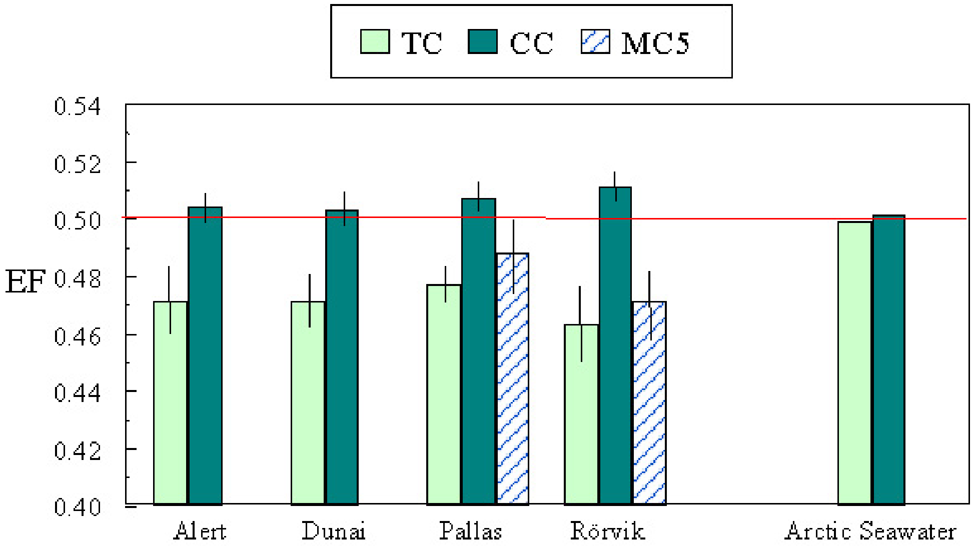

**FIGURE 4.** Enantiomer fractions of TC, CC, and MC5 in arctic air and seawater. Vertical lines indicate standard deviations. Air data from Bidleman et al.[73], water data from Jantunen and Bidleman[18].

## Air-Water Exchange

The gas exchange of HCH isomers in the Great Lakes undergoes seasonal changes due to variations in water temperature, air concentrations, and stratification of the water column[11]. In the western Arctic Ocean and the Bering and Chukchi seas, the net exchange direction of α-HCH has reversed in response to declining atmospheric concentrations, from deposition in the 1980s to volatilisation in the 1990s[17,52,74,75]. Currently, α-HCH is close to air-water equilibrium in the eastern Arctic Ocean and γ-HCH is undergoing net deposition[19]. One reason for this might be the replacement of technical HCH by lindane, which is currently used in several countries.

Volatilisation of α-HCH from water bodies can be followed by the appearance of nonracemic α-HCH in the air boundary layer above the water. Air samples were depleted in (+)α-HCH above the unfrozen areas of the western[17] and eastern[19] Arctic Ocean and in (−)α-HCH above the Bering-Chukchi seas[17], mirroring the enantiomer depletions in the surface waters of these regions. The α-HCH in air over ice-covered portions of the Arctic Ocean were close to racemic, even though nonracemic α-HCH was found in the underlying water. Thus, the racemic signature of α-HCH found in continental regions[14,39] is also seen over the Arctic Ocean when gas exchange is hindered.

In Lake Ontario, (+)α-HCH is preferentially depleted (EF = 0.459)[11]. The enantiomer profiles in air over the lake showed seasonal changes which followed the trend of water/air fugacity ratios. In fall and spring, fugacity ratios were less than 1.0 (net deposition) and EFs were close to 0.50 (racemic) indicating that most of the α-HCH came from distant transport. In summer, fugacity ratios were greater than 1.0 (net volatilisation) and EFs in air declined to as low as 0.476 as nonracemic α-HCH volatilised from the lake.

Air-water exchange of α- and γ-HCH was investigated in the southern Baltic Sea during the summer of 1997 and the winter of 1998[20]. The HCHs were close to partitioning equilibrium, but net fluxes varied over time, especially during the summer. Variations in the concentration fraction $F_{\alpha}$ = α-HCH/(α-HCH + γ-HCH) in the air were related to transport direction, with lower fractions (enrichment of γ-HCH) associated with airflow from central Europe. The EFs of α-HCH in air varied from 0.455 to 0.485 and were generally lower when the air passed over the southern Baltic Sea, where the EF in water averaged 0.445 compared to transport from the north. A linear relationship ($r^2 = 0.45$, $p < 0.035$) was found between $F_{\alpha}$ and EF.

An equation for estimating the contribution of a chiral compound from two sources, A and B, in an A-B mixture was derived in terms of the ER values of the compound in A, B, and the mixture[76]. The relationship is simplified by using EF instead of ER[41]:

$$F_a = (EF_m - EF_b)/ (EF_a - EF_b) \quad (1)$$

where $EF_a$, $EF_b$, and $EF_m$ are the EF values for A, B, and the mixture, respectively. The estimated volatilisation contribution of α-HCH to the air over Lake Ontario in a situation where $EF_a$ = 0.459, $EF_b$ = 0.500, and $EF_m$ = 0.476 was 58%[76]. The fraction of sea-derived α-HCH in air over the southern Baltic was estimated to be 53–66% in summer and 0–35% in winter[20].

The enantiomeric composition of α-HCH has also been determined in precipitation. Rain samples collected on the shore of Lake Ontario contained racemic α-HCH, even in summer when nonracemic α-HCH was found in air over the lake[11]. The authors suggested that rain scavenged racemic α-HCH from above the boundary layer and did not have time to re-equilibrate with the nonracemic α-HCH over the lake. However, the EF in rain collected from shore may not be influenced by air over the water but may instead be dominated by transport of fresh technical HCH[77]. Precipitation was collected at the west coast of the Wadden Sea, Germany, in a location where the prevailing winds were from the sea[77]. EFs of α-HCH ranged from 0.468–0.505 and showed a seasonal trend of lower values (more nonracemic) in the warmer months of the year. Thus, the EF in rain was probably influenced by α-HCH volatilising from the sea (average EF in water = 0.460), and the apparent contribution of α-HCH from the sea to the rain in summer was estimated to be 29–73%.

EFs ranging from 0.478 to 0.494 were found for α-HCH in rain collected from Gotland Island in the southern Baltic[20]. In contrast to the German study, EFs here were more nonracemic in the winter than in summer. However, only three rain samples were collected in each season and these were integrated over collection times of 8–32 days. The EFs in air samples taken in the region showed a strong influence of transport direction, as noted above.

Hühnerfuss et al.[50] demonstrated in laboratory experiments that the dehydrochlorination products of HCHs, the isomeric PCCHs, could also undergo gas exchange. The two isomers appear to have substantially different air/water partition coefficients (Henry's law constants). When air and water were equilibrated in a flask (relative volumes unspecified) for 3 weeks, 99% of the γ-PCCH partitioned from water into the air, whereas transfer of β-PCCH to air was only minor.

## CONCLUSIONS AND SUGGESTIONS FOR FUTURE RESEARCH

The examples presented here illustrate the application of chiral compounds as tracers for following biotic degradation in soil and water, and air-surface exchange of chiral OCPs. Studies involving enantiomers would be enhanced by further work in several areas.

Analytical methods for chiral xenobiotics have been extensively reviewed[31,32,33]. Regarding capillary GC methods, there are presently a limited number of commercial columns which can resolve enantiomers of chiral pesticides. Most chiral columns contain nonbonded phases, an exception being Chirasil-Dex, an immobilised permethylated β-cyclodextrin phase. Nonbonded phases tend to bleed and cause high background signals in GC-ECD and GC-MS. Higher temperature separations, improved signal-to-noise, and longer column life could be achieved with bonded-phase chiral columns. A major problem with chiral columns has been variability in stationary phase composition. The cyclodextrins used for chiral stationary phases are often not completely derivatised, and small changes in the percent derivatisation can greatly affect chiral separation[78,79,80,81]. High purity and reproducibility in the preparation of derivatised cyclodextrins are essential for reliable analytical results[31,33,78,79,80,81].

Studies of the rates and mechanisms of enantioselective metabolism in soil and water are sparse, and there are very few cases in which attempts have been made to identify/isolate the microbial communities or enzymes responsible. Examples of the latter are the studies in Lewis et al.[47], Garrison et al.[48], Hühnerfuss et al.[50], and Ludwig et al.[82]. An initial study has not shown enantioselectivity of plant enzymes in the degradation of o,p´-DDT[48]; however, this should not discourage further investigations of this type.

Is it possible that abiotic mechanisms contribute to observed enantioselectivity in soils? Studies have shown selective adsorption of D- or L-amino acids by calcite[83], quartz[84], and bentonite clay[85] (although the latter is controversial[86,87,88]). There is a need to investigate enantioselective sorption of OCPs by soil constituents such as clay minerals and humics. Indeed, Lewis et al.[47] stated that "...many soils have their genesis with living matter and their organic components may retain chiral centres capable of selectively binding specific enantiomers. Enantioselective sorption to soil organics, if it occurs, could affect the availability of various enantiomers for microbial transformation." Chiral surfactants such as long-chain fatty acids, amino acids, and their methyl esters form monolayers at the air-water interface and chiral discrimination is enhanced by bivalent cations ($Ca^{+2}$, $Pb^{+2}$, $Zn^{+2}$)[89,90,91,92]. Could chiral interactions at the air-water interface lead to discrimination in the gas exchange of enantiomers?

Laboratory or field experiments should be carried out to check whether the enantiomer signatures of chemicals volatilising from soil and water do in fact match the patterns in these reservoirs, and are not affected by selective binding of one enantiomer. To use enantiomers as tracers of volatilisation on a regional scale and incorporate EF data into regional models, it is necessary to determine the spatial and temporal distribution of chiral OCP residues in soil and water.

## NOTE ADDED IN PROOF

Recently Mattina et al.[93] reported a study of chlordane isomers and enantiomers transferred from soil to vegetable crops and overlying air at the Connecticut Agricultural Experiment Station. Zucchini showed a 2.5- to 8-fold enhancement of chlordane and nonachlor isomers in the root relative to the soil. Changes in the relative concentrations of chlordane enantiomers were found among bulk soil and various compartments of plants, indicating that enantioselective processes were in effect. EFs of TC (0.46) and CC (0.53) in air at 0.6 m height were most similar to those in soil (0.45 and 0.55, respectively for TC and CC) and approached background air EFs (0.48 and 0.52, respectively) with increasing height above the soil. The EFs in leaves and fruit peel of cucumber growing within the 0.6-m zone ranged from 0.30–0.37 for TC and 0.48–0.52 for CC, suggesting that enantioselective processes within the plant, rather than air-plant exchange, dominated the observed enantiomer signatures.

## ACKNOWLEDGEMENTS

This work was funded in part by the Canadian Toxic Substances Research Initiative (TSRI), a research program managed jointly by Health Canada and Environment Canada, under Project #11. Work in the Canadian Arctic was supported in part by Indian and Northern Affairs Canada under the Northern Contaminants Program. P.A. Helm's work was supported in part by a scholarship from the National Science and Engineering Research Council of Canada.
